# Health system and treatment regimen determinants of delayed first-dose antibiotic administration in hospitalized adults in Uganda

**DOI:** 10.21203/rs.3.rs-9003231/v1

**Published:** 2026-03-27

**Authors:** Nsereko Mansoor Kalimunda, Hood Ibanda, Denis Omali, Isaac Magulu Kimbowa, Ronald Kiguba

**Affiliations:** Department of Pharmacology and Therapeutics, Makerere University College of Health Sciences; Department of Pharmacology and Therapeutics, Makerere University College of Health Sciences; Directorate of Research and Grants, Kabale University Isaac; Department of Pharmacology and Therapeutics, Makerere University College of Health Sciences; Department of Pharmacology and Therapeutics, Makerere University College of Health Sciences

**Keywords:** Antibiotic initiation, time-to-antibiotics, health system factors, antimicrobial stewardship, hospital inpatients, length of stay, Uganda

## Abstract

**Background:**

Delayed first-dose antibiotic administration among hospitalized adults partly contributes to poor treatment outcomes. However, the operational drivers of such delays remain poorly characterized in low-resource settings. We sought to delineate the possible operational drivers of these delays, and suggest potential healthcare in Uganda.

**Methods:**

We conducted a cross-sectional study of 606 adult inpatients with suspected or confirmed bacterial infections at two public health facilities in Uganda. Delayed administration/initiation was defined as administration of the first antibiotic dose 〿 48 hours after prescription. Data were obtained through interviewer-administered questionnaires and medical record review. Data analysis was performed using multivariable logistic regression.

**Results:**

Among 606 participants, 36 (6.0%) experienced delayed antibiotic administration. Delays were strongly associated with health system determinants, such as waiting for more than 12 hours before initial consultation (aOR 5.35, 95% CI 2.43–11.78), lack of convenient facility hours (aOR 5.02, 95% CI 2.41–10.44) and inadequate medical staffing (aOR 2.37, 95% CI 1.06–5.27). Treatment complexity was a key clinical driver, where prescription of three antibiotics but not monotherapy, was associated with substantially higher odds of delay (aOR 5.69, 95% CI 1.85–17.52). Generally, patient demographic characteristics were not associated with delay. Patients reporting inadequate household income had lower odds of delay (aOR 0.29, 95% CI 0.12–0.67).

**Conclusions:**

Although delayed first-dose antibiotic administration was uncommon, it was driven by modifiable health system inefficiencies and regimen complexity. Targeted operational interventions like improving triage processes, extending facility hours, ensuring adequate medical staffing and streamlining multi-drug regimens reduce such delays. This could consequently support effective antibiotics use towards intended treatment outcomes in resource-constrained settings.

## Introduction

The first dose of an antibiotic is a critical intervention in the management of acute bacterial infections (1). The goal is not just to give antibiotics, but to give the right antibiotic as soon as possible after the need is identified. In conditions like sepsis, severe pneumonia, and meningitis, every hour of delay in antibiotic administration is associated with a significant increase in mortality and morbidity (2–4). The principle of “timeliness” in antibiotic initiation therefore remains cornerstone to effective antimicrobial therapy as guided by international guidelines for sepsis and severe infection (1, 5). However, in low- and middle-income countries (LMICs), health system constraints often make timely administration a major challenge (6, 7).

For example, a study in a Malawian referral hospital found that bottlenecks/challenges in triage and consultation were primary drivers of delays in administering the first antibiotic dose (8). Similarly, research in Tanzania reported that a significant proportion of inpatients experienced clinically important delays in antibiotic initiation (9). These system-level failures including drug stock-outs, understaffing and inefficient patient flow are compounded by patient-related bottlenecks like financial constraints and delays in seeking care, creating a perfect storm for suboptimal treatment initiation (6, 10).

Consequently, delayed antibiotic first-dose initiation is likely associated with prolonged infection and clinical deterioration, and hence longer hospital stays, increased healthcare costs, and a greater burden on already strained systems(11). Furthermore, delayed or inadequate initial therapy is a recognized driver of antimicrobial resistance (AMR) (10, 12). When treatment is delayed, therapeutic drug concentrations are hardly attained. This is partly due to potential time-dependent increase in bacterial load. This cascade of events (delayed antibiotic initiation-increased bacterial load-subtherapeutic drug concentrations) potentially contributes to AMR, which complicates antimicrobial therapy and worsens treatment outcomes (10, 13).

In Uganda, while some studies have documented high rates of antibiotic prescription, the focus has rarely been on the timeliness of administration after the clinical decision is made (14). Existing research has highlighted financial barriers and prescribing patterns but lacks a granular analysis of the specific intra-hospital bottlenecks that cause delays after a patient has been admitted with a written prescription (14). For example, the extent to which factors like long waiting times for consultation, complex multi-drug regimens, nursing shortages, or pharmacy dispensing logs contribute to delays in Ugandan tertiary care facilities remains unknown. We sought to delineate the specific, modifiable health system factors that are essential in improving antibiotic administration in adult inpatients. Findings reported here could support targeted interventions, such as antimicrobial stewardship programs and operational improvements that are designed to optimize patient outcomes in Uganda and similar resource-constrained settings.

## Methods

### Study aim

The current study aimed to identify both health system and treatment regimen determinants of delayed first-dose antibiotic initiation in adult inpatients in Uganda.

### Study design

We conducted a cross-sectional study from April 2023 to June 2023 among adult patients admitted with suspected of confirmed bacterial infections at two public health facilities in Kampala, Uganda: Kiruddu National Referral Hospital (tertiary care, ~ 200 beds) and Kisenyi Health Centre IV (secondary care, ~ 50 beds). These sites were selected to represent different levels of Uganda’s hierarchical healthcare system (15).

### Study setting

Uganda’s health system follows an integrated structure comprising of approximately 6,937 health facilities, where 45% are public-owned, 40% are private for-profit (PFP), and 15% are private-not-for-profit (PNFPs) (15). The public health system is referral-based and provides free services at all levels of delivery (15). The composition of public health facilities included two national referral hospitals, 16 regional referral hospitals, 47 general hospitals, 166 level IV health centers, 962 level III health centers, 1,321 level II health centers, and 1,558 clinics at the time of conducting this study (15).

Kiruddu National Referral Hospital is one of the largest hospitals in Uganda, previously part of Mulago Hospital Complex. It offers services in internal medicine, burns, plastic surgery, radiology, and palliative care, and serves as a teaching hospital for Makerere University College of Health Sciences. Kisenyi Health Centre IV is managed by Kampala Central City Authority and provides secondary care services.

### Characteristics of study participants

Adult patients aged 18 years or older and hospitalized with suspected or confirmed bacterial infections based on clinical or laboratory diagnosis were enrolled. All participants received systemic antibiotics during admission. We excluded critically ill patients, patients without documented medical history and those receiving topical and prophylactic antibiotics.

### Sample Size determination

We determined the sample size using the Kish Leslie formula for a single population proportion: N = (Zα^2^ * P * (1-P)) / δ^2^. We used a conservative prevalence (P) of 50% for delayed antibiotic initiation to maximize the sample size. With zα = 1.96 (95% confidence level) and δ = 0.05 (5% margin of error), the calculation yielded a minimum of 384 participants. After adding 10% for non-response, the target was 422. However, as we employed a two-stage sampling strategy involving proportionate sampling from two facilities of different sizes, we anticipated a design effect. A design effect of 1.5 was applied to adjust for the loss of efficiency compared to simple random sampling. The adjusted sample size was calculated as 422 * 1.5 = 633.

Finally, to achieve proportional allocation from the two study sites based on their average monthly admission rates (Kiruddu NRH: 3000; Kisenyi HCIV: 600), the sample size was fine-tuned. To ensure a practical and implementable sampling interval at both sites, the sample size was adjusted to 606, which allowed for a consistent sampling interval of 6 at both facilities while maintaining a strong representation of the source population as presented below.

KisenyiHCIV:(600/3600)*606=101.

KirudduNRH:(3000/3600)*606=505.

### Sampling procedure

A two-stage sampling approach was employed. In the first stage, two public health facilities Kiruddu National Referral Hospital and Kisenyi Health Centre IV were purposively selected to represent different levels of care within Uganda’s public health system. In the second stage, a systematic sampling method was used to recruit individual participants within each facility. The sample size was allocated to each facility using probability proportionate to size (PPS), based on their average monthly admission rates (Kiruddu NRH: 3000; Kisenyi HCIV: 600). This PPS calculation determined that 505 participants would be recruited from Kiruddu NRH and 101 from Kisenyi HCIV, ensuring the sample was representative of the patient volume at each site. The sampling interval was then calculated separately for each facility by dividing the estimated number of eligible patients by the required sample size, resulting in an interval of 6 at both sites. The first participant on each ward was selected using simple random sampling (by picking a number between 1 and 6), after which every 6th eligible patient was invited to participate. If a selected patient was ineligible, the next consecutive eligible patient was enrolled.

### Data Collection Tools

An interviewer-administered questionnaire that was used by a previous related study (14) was adopted by the current study with permission and minimal modifications modified to investigate delays in antibiotic initiation following prescription. The questionnaire’s face and content validity were rigorously assessed by a multidisciplinary team comprising a pharmacologist, pharmacist, epidemiologist and public health specialist. The tool was subsequently piloted on 10 adult in-patients at Kisenyi Health Centre IV. Feedback regarding question wording and clarity was incorporated into the final instrument, and data from this pilot phase were excluded from the final analysis. The final questionnaire comprised four comprehensive sections;

Section A: Facility Identifiers, Screening, and Consent.

Section B: Socio-demographic Characteristics.

Section C: Clinical and Temporal Data.

Section D: Health System Factors.

### Variables and Definitions

The primary outcome of this study was delayed first-dose antibiotic initiation. This was defined as the administration of the first antibiotic dose more than 48 hours after the time of prescription (T = 0). The outcome was measured using data extracted from patient medical records, which documented the precise date and time of the antibiotic prescription and the corresponding date and time of the first administered dose. This temporal data, collected under Section C of the data collection tool, allowed for the exact calculation of the time elapsed between prescription and administration for each patient.

Independent variables investigated for their association with this delay were categorized into three groups: Patient-related factors: Age, sex, marital status, place of residence, education level, and self-reported household income status. Treatment-related factors: The complexity of the initial antibiotic regimen (categorized as 1, 2, or 3 + antibiotics) and the primary disease category. Health system-related factors: Time to initial consultation (> 12 hours versus ≤ 12 hours), facility accessibility, lack of convenient facility hours, inadequate staffing, and the need for external antibiotic procurement.

### Data collection and management

Study data was collected using a validated interviewer-administered questionnaire, and supplemented by medical record reviews. Four trained research assistants (three at Kiruddu NRH, one at Kisenyi HCIV) screened patients for eligibility, obtained written informed consent at admission, and administered the questionnaires (the entire processing taking about 15 minutes). Research assistants reviewed patients’ medical files to extract clinical and temporal data. Completed questionnaires were stored in a lockable safety cabinet and collected weekly for review by the Principal Investigator. Data collection was finalized and verified against medical records at discharge or death.

A comprehensive data management process was implemented to ensure data quality. Daily data review was performed to identify and correct any missing variables. During data cleaning, files with major missing variables were removed from the analysis. Data were double-entered and validated using EpiData software (version 4.4.2.1) to ensure accuracy. All data were then imported into STATA (version 17) for cleaning and statistical analysis.

### Data analysis

Data were summarized using descriptive statistics where continuous variables were summarized as means and standard deviations, while categorical data were summarized as frequencies and proportions. The prevalence of delayed first-dose antibiotic initiation was calculated as the proportion of inpatients who experienced a delay (> 48 hours) out of the total study sample. Bivariate analyses between independent variables and the outcome were conducted using Chi-square tests, with results reported as crude odds ratios (OR) with 95% confidence intervals (CI). Variables with a p-value < 0.2 in the bivariate analysis and those deemed clinically relevant were included in a multivariable logistic regression model to identify independent predictors. The results of the final model are presented as adjusted odds ratios (aOR) with 95% CIs. Confounding and effect modification were assessed during the model-building process. A p-value of < 0.05 was considered statistically significant for all analyses.

## Results

### Participant socio-demographics characteristics

The study included 606 adult in-patients from two selected health facilities in Kampala, Uganda, majority (83%, n = 505) of participants recruited from Kiruddu National Referral Hospital. The participant population had a median age of 34 years (IQR: 25–52), with a mean age (standard deviation) of 35.8 (11.4). Women comprised 66% (n = 400) of the sample. Most participants were married (62%, n = 375), over four-fifth (80%, n = 500) of participants had at least some formal education and (29%, n = 173) had completed primary education. Two-thirds of participants (65%, n = 398) reported their household income was inadequate to meet basic needs, most participants resided in suburban areas (49%, n = 299) as detailed in Table 1.

### Participant clinical characteristics

The majority of participants (505/606, 83.3%) were recruited from Kiruddu National Referral Hospital. Most patients (570/606, 94%) experienced no delay in treatment. Majority of adult inpatients (167/606, 27.6%) had suspected gastrointestinal bacterial infections. In terms of treatment intensity 243 patients received one antibiotic (monotherapy), 278 received two antibiotics, and 85 received three antibiotics as detailed in Table 2.

### Antibiotic use in adult in-patients, by regimen type

Cephalosporins were the most frequently prescribed antibiotic class overall and the dominant choice for monotherapy, accounting for nearly half of all single-antibiotic regimens. Penicillins, while the second most prescribed class overall, were rarely used as monotherapy and were predominantly employed in combination regimens. Nitronitroimidazoles were also frequently used as a single agent. In contrast, aminoglycosides were exclusively used in combination therapy, with their use distributed between dual and triple regimens. A key finding was the critical role of the “Other” category (e.g., vancomycin), which became the most prescribed class within triple-therapy regimens, indicating its use for severe or complex infections. The distribution of antibiotic classes across different therapy intensities is detailed in Table 3.

### Prevalence of delayed first antibiotic dose among study adult inpatients

This study examined the prevalence of delays in administering the first antibiotic dose to adult inpatients in Kampala, Uganda. It found that 6% (36/606) of patients experienced a significant delay (≥ 48 hours), while 94% received timely treatment (within 48 hours). The prevalence of delay varied across different patient groups. A higher prevalence of delay was observed among patients prescribed more complex antibiotic regimens: 2.1% (5/243) for one antibiotic, 6.1% (17/278) for two antibiotics, and 11.8% (10/85) for three antibiotics. The prevalence of delay also varied by financial status: 18.2% (2/11) in patients with a financial surplus, 8.1% (16/197) in those with adequate income, and 4.5% (18/398) in those with inadequate income. While rural and suburban residents had a numerically higher prevalence of delay (7.1% and 6.7%, respectively) compared to urban patients (2.4%), the treating facility and the patient’s primary disease category showed minimal variation in the prevalence of administration delays. These descriptive findings warranted further investigation via multivariable analysis to identify independent predictors.

### Factors associated with delayed first dose antibiotic initiation among adult inpatients

After adjusting for potential confounders in the multivariable logistic regression model, several factors were independently associated with delayed antibiotic initiation. Demographic characteristics including age, sex, and marital status showed no significant association with delay. Additionally, health facility accessibility and the need for external antibiotic procurement were not significant predictors in the final model. The factors retained in the final model are presented in Table 5.

## Discussion

Our study found a low prevalence (6%) of delayed first-dose antibiotic initiation from the time of prescription among adult inpatients in two Ugandan tertiary care facilities. This rate is notably lower than those reported in a similar study from Tanzania (31%) (9), a discrepancy that may be partly explained by differences in study settings and definitions of delay. This finding suggests that the post-prescription administration process in these Ugandan facilities is highly efficient for the vast majority of patients. However, within this context of overall efficiency, our multivariate analysis identified critical and modifiable bottlenecks associated with the delays that do occur.

The most robust predictors of delay were structural health system inefficiencies. Patients who waited over 12 hours for their initial consultation had over five times the odds of a delayed antibiotic dose. This finding aligns with research from Malawi, where bottlenecks in triage and consultation were primary drivers of treatment delays (8). Furthermore, the lack of convenient facility hours increased the odds of delay more than fivefold. In Uganda, where many public facilities have limited operational hours, patients presenting at night or on weekends may experience critical hold-ups until services resume. Inadequate staffing was another independent predictor, reinforcing findings from multi-country studies linking lower nurse-to-patient ratios to missed care and longer time-to-treatment (9, 16, 17). Collectively, these factors highlight that when delays do occur, they are not random failures but are deeply rooted in specific operational and resource constraints (18), underscoring the critical importance of time to appropriate therapy (11, 19).

A key clinical finding was that the complexity of the initial antibiotic regimen was a strong, dose-dependent predictor of delay. Patients prescribed a three-antibiotic regimen had nearly six times the odds of delay compared to those on monotherapy. This suggests that logistical challenges such as the time required to procure, prepare, and administer multiple medications from potentially different sources-introduce a significant barrier to timely initiation (8, 20). The burden of these complex regimens is further illuminated by their specific composition; our data reveal that triple-therapy was distinctly characterized by a heavy reliance on antibiotics from the ‘Other’ category such as glycopeptides (e.g., vancomycin) which constituted over a quarter (27.1%) of all prescriptions in three antibiotics regimens. These agents are typically reserved for severe, multidrug-resistant, or nosocomial infections and often require special handling, or specific monitoring (10) (21). Furthermore, such reserve antibiotics are frequently less readily available in hospital formularies or require special approval processes, creating procurement bottlenecks that can critically impact time-to-therapy (11, 19). Therefore, the delay associated with complex regimens is likely not just a function of the number of drugs, but also a consequence of the specific, logistically complex agents that define these advanced treatment protocols.

A counterintuitive yet important finding was that patients self-reporting inadequate household income had 71% lower odds of experiencing delay than those with adequate income. This contrasts with much of the global literature linking poverty to healthcare delays (22) but may be explained by the protective effect of Uganda’s tax-funded public health system, which provides free services and may buffer financial barriers for the poorest patients (15). An alternative explanation is potential clinical prioritization, where healthcare providers may unconsciously perceive poorer patients as being at higher acuity or having fewer alternative care options, leading to expedited processes for them once within the system. This paradoxical finding warrants further qualitative investigation to understand the roles of provider perception and decision-making.

The consequences of delayed antibiotic initiation are well-documented and extend beyond the individual patient. Even in a small proportion of patients, such delays can lead to treatment failure, prolonged hospitalization, and increased healthcare costs (11). Furthermore, inadequate or delayed antimicrobial therapy is a key driver of antimicrobial resistance (AMR) (10). In a low-resource setting with a limited arsenal of affordable antibiotics, practices that contribute to AMR, including delays that compromise efficacy, pose a significant public health threat (14), as demonstrated by trials showing increased mortality with treatment delays in similar settings (23).

Our findings point to several actionable areas for intervention. To address the health system barriers, operational changes such as extending facility hours, improving triage systems to reduce consultation waits, and investing in adequate staffing are critical. To mitigate the risk associated with complex regimens, clinical interventions could include developing standardized protocols and pre-packaged order sets for common multi-drug regimens to streamline their preparation and administration. These multi-faceted strategies are directly aligned with the goals of Uganda’s National Action Plan on antimicrobial resistance (7) and are essential for strengthening health systems and improving patient outcomes in resource-constrained settings, as called for in recent Ugandan scoping reviews on stewardship (24).

Despite the critical relevance of our findings to public health practice, the current study had some limitations, some of which were inevitable and out of the study context. First, the current study did not report microbiological culture results for participants that were identified as having bacterial infection. This was partly due to common practice of empirical treatment in the Ugandan setting, yet mistreatment could directly impact on length of stay in the hospital. Secondly, the cross-sectional design of this study limited the exploration of potential data regarding actual treatment outcomes such as re-infection and relapse. Future studies should consider a longitudinal design and actual bacterial culture design to support bacterial strain-poor treatment outcome causal link. Such a study could draw attention to particular microbes and antibiotic pairs that require maximum attention regarding empirical drug use and urgent administration of antibiotics.

## Conclusions

While delays in antibiotic initiation after prescription were uncommon in this Ugandan setting, they were strongly associated with modifiable health system inefficiencies and complex treatment regimens. Interventions targeting process optimization such as extending facility hours, improving triage and staffing, and implementing streamlined protocols for multi-drug regimens are essential to minimize delays and their associated risks. The protective effect of lower income suggests Uganda’s public health system may buffer financial barriers to timely care, a finding that merits further exploration. These findings highlight specific targets for health system strengthening and antimicrobial stewardship initiatives in similar resource-constrained environments. This could consequently support effective antibiotics use towards intended treatment outcomes in resource-constrained settings.

## Figures and Tables

**Table 1:** Socio-demographic characteristics of study adult in-patients

Characteristics	Frequency	Percentage %
Sex		
Male	206	34
Female	400	66
Age (years) groups		
18–24	137	23
25–34	177	29
35–44	94	16
45+	198	33
Place of residence		
Urban	123	20
Suburban	299	49
Rural	182	30
Homeless	2	01
Marital status		
Single	104	17
Married	375	62
Divorced/separated/widowed	127	21
Level of education		
Advanced/college	53	9
Secondary	274	45
Primary	173	29
No formal education	106	18
Household income status		
**Surplus** (≥ 50% above needs)	11	2
**Adequate** (covers needs)	197	33
**Inadequate** (cannot cover needs)	398	66

**Table 2:** Clinical characteristics that determined antibiotic use in study adult in-patients

Category	Total	Percentage (%)
Disease State (ICD-10 Code)		
Gastrointestinal (K63.9, E34.9)	167	27.6
Obstetrics/Gynecology (O99.8)	151	24.9
Respiratory (J98.9)	98	16.2
Cardiovascular (I51.9)	73	12.0
Genitourinary (N28.9)	65	10.7
Skin (L98.9)	35	05.8
Other	17	02.8
Delay Status		
0–48 hrs (No delay)	570	94.0
49 + hrs (Delayed)	36	06.0
Number of Patients by initial antibiotic Regimen		
1 antibiotic	243	40.1
2 antibiotics	278	45.9
3 antibiotics	85	14.0

Key: hrs- hours, ATC- Anatomical Therapeutic Chemical classification, ICD- International Classification of Diseases.

**Table 3:** Antibiotic use in adult inpatients, by regimen type

Antibiotic Class (ATC Code)	Total Times Prescribed (%)	Distribution within 1 antibiotic regimen (N = 243) (%)	Distribution within 2 antibiotics regimens (N = 556) (%)	Distribution within 3 antibiotics regimens (N = 255) (%)
Cephalosporins (J01D)	385 (36.5)	121 (49.8)	197 (35.4)	67 (26.3)
Penicillin (J01C)	241 (22.9)	28 (11.5)	149 (26.8)	64 (25.1)
Fluoroquinolones (J01MA)	164 (15.6)	36 (14.8)	96 (17.3)	32 (12.5)
Nitronitroimidazoles (J01XD)	99 (9.4)	43 (17.7)	47 (8.5)	9 (3.5)
Macrolides (J01F)	44 (4.2)	11 (4.5)	26 (4.7)	7 (2.7)
Aminoglycosides (J01G)	12 (1.1)	0 (0.0)	5 (0.9)	7 (2.7)
Other	109 (10.3)	4 (1.6)	36 (6.5)	69 (27.1)
Total Prescriptions across regimen (N)	1,054(100)	243(100)	556(100)	255(100)

The “Other” antibiotic category primarily comprised glycopeptides (e.g., vancomycin), tetracyclines (e.g., doxycycline), and sulfonamides (e.g., sulfamethoxazole-trimethoprim).

**Table 4:** Prevalence of delayed first-dose antibiotic initiation (from time of prescription) among adult inpatients

Factor	Total N (%)	Not Delayed (0–48hrs) N (%)	Delayed (> 48hrs) N (%)
Facility			
Kiruddu NRH	505 (83.3)	472 (93.5)	33 (6.5)
Kisenyi HCIV	101 (16.7)	98 (97.0)	3 (3.0)
Disease State (ICD-10 Code)			
Gastrointestinal (K63.9, E34.9)	167 (27.6)	160 (95.8)	7 (4.2)
Obstetrics/Gynecology (O99.8)	151 (24.9)	143 (94.7)	8 (5.3)
Respiratory (J98.9)	98 (16.2)	**85 (86.7)**	**13 (13.3)**
Cardiovascular (I51.9)	73 (12.0)	70 (95.9)	3 (4.1)
Genitourinary (N28.9)	65 (10.7)	62 (95.4)	3 (4.6)
Skin (L98.9)	35 (5.8)	34 (97.1)	1 (2.9)
Other	17 (2.8)	16 (94.1)	1 (5.9)
Financial Status			
Surplus	11 (1.8)	9 (81.8)	2 (18.2)
Adequate	197 (32.5)	181 (91.9)	16 (8.1)
Inadequate	398 (65.7)	380 (95.5)	18 (4.5)
Patient’s Residence			
Urban	123 (20.3)	120 (97.6)	3 (2.4)
Suburban	299 (49.3)	279 (93.3)	20 (6.7)
Rural	182 (30.0)	169 (92.9)	13 (7.1)
Homeless	2 (0.3)	2 (100.0)	0 (0.0)
Number of Patients by initial antibiotic Regimen			
1 antibiotic	243(40.1)	238(97.9)	5(2.1)
2 antibiotics	278(45.9)	261(93.9)	17(6.1)
3 antibiotics	85(14.0)	75(88.2)	10(11.8)

Delay was calculated from the time the antibiotic was prescribed to the time the first dose was administered

**Table 5:** Factors associated with delayed first dose antibiotic initiation

Factor	Category	Adjusted Odds Ratio (aOR)	95% CI	p-value
Health System Factors				
Time to consultation	> 12 hours (Ref: ≤12 hours)	5.35	2.43–11.78	0.001
Convenient facility hours	No (Ref: Yes)	5.02	2.41–10.44	0.001
Adequate staffing	No (Ref: Yes)	2.37	1.06–5.27	0.035
Treatment-Related Factor				
Initial antibiotic regimen	2 antibiotics (Ref: 1 antibiotics)	2.93	1.05–8.19	0.040
	3 antibiotics (Ref: 1 antibiotics)	5.69	1.85–17.52	0.002
Patient-Related Factor				
Household income status	Inadequate (Ref: Adequate*)	0.29	0.12–0.67	0.006

* The reference group for Household Income Status (‘Adequate’) combines participants who reported ‘Adequate’ and ‘Surplus’ income. Abbreviation: Ref-Reference category.

## Data Availability

All data generated or analysed during this study are included in this published article. Any other data that could be of interest to the reader can be accessible by contacting the corresponding author on reasonable request.

## Abbreviations

AMR: Antimicrobial resistance
aOR: Adjusted odds ratio
CI: Confidence interval
HCIV: Health center IV
LMICs: Low-and middle-income countries
NRH: National referral hospital
OR: Odds ratio
PPS: Probability proportionate to size
